# Supplementary material for: Resolving the full spectrum of human genome variation using Linked-Reads
Source: Genome Res. 2019 Apr;29(4):635–45. doi: 10.1101/gr.234443.118 (PMC6442396; doi:10.1101/gr.234443.118)
Supplement: Supplemental Material [file supp_gr.234443.118_Supplemental_longranger-2.2.zip › longranger-2.2.tar.bz2!/lr2.2-public/lib/stan/cmdstan/stan/doc/api/html/structstd_1_1numeric__limits_3_01stan_1_1agrad_1_1fvar_3_01_t_01_4_01_4-members.html]

Stan: Member List


|  |  |
| --- | --- |
|  | Stan  2.5.0  probability, sampling & optimization |


- Main Page
- Related Pages
- Namespaces
- Classes
- Files

- Class List
- Class Index
- Class Hierarchy
- Class Members

All Classes Namespaces Files Functions Variables Typedefs Enumerations Enumerator Friends Macros Pages

- std
- numeric\_limits< stan::agrad::fvar< T > >

std::numeric\_limits< stan::agrad::fvar< T > > Member List

This is the complete list of members for std::numeric\_limits< stan::agrad::fvar< T > >, including all inherited members.

|  |  |  |
| --- | --- | --- |
| denorm\_min() | std::numeric\_limits< stan::agrad::fvar< T > > | inlinestatic |
| digits | std::numeric\_limits< stan::agrad::fvar< T > > | static |
| digits10 | std::numeric\_limits< stan::agrad::fvar< T > > | static |
| epsilon() | std::numeric\_limits< stan::agrad::fvar< T > > | inlinestatic |
| has\_denorm | std::numeric\_limits< stan::agrad::fvar< T > > | static |
| has\_denorm\_loss | std::numeric\_limits< stan::agrad::fvar< T > > | static |
| has\_infinity | std::numeric\_limits< stan::agrad::fvar< T > > | static |
| has\_quiet\_NaN | std::numeric\_limits< stan::agrad::fvar< T > > | static |
| has\_signaling\_NaN | std::numeric\_limits< stan::agrad::fvar< T > > | static |
| infinity() | std::numeric\_limits< stan::agrad::fvar< T > > | inlinestatic |
| is\_bounded | std::numeric\_limits< stan::agrad::fvar< T > > | static |
| is\_exact | std::numeric\_limits< stan::agrad::fvar< T > > | static |
| is\_iec559 | std::numeric\_limits< stan::agrad::fvar< T > > | static |
| is\_integer | std::numeric\_limits< stan::agrad::fvar< T > > | static |
| is\_modulo | std::numeric\_limits< stan::agrad::fvar< T > > | static |
| is\_signed | std::numeric\_limits< stan::agrad::fvar< T > > | static |
| is\_specialized | std::numeric\_limits< stan::agrad::fvar< T > > | static |
| max() | std::numeric\_limits< stan::agrad::fvar< T > > | inlinestatic |
| max\_exponent | std::numeric\_limits< stan::agrad::fvar< T > > | static |
| max\_exponent10 | std::numeric\_limits< stan::agrad::fvar< T > > | static |
| min() | std::numeric\_limits< stan::agrad::fvar< T > > | inlinestatic |
| min\_exponent | std::numeric\_limits< stan::agrad::fvar< T > > | static |
| min\_exponent10 | std::numeric\_limits< stan::agrad::fvar< T > > | static |
| quiet\_NaN() | std::numeric\_limits< stan::agrad::fvar< T > > | inlinestatic |
| radix | std::numeric\_limits< stan::agrad::fvar< T > > | static |
| round\_error() | std::numeric\_limits< stan::agrad::fvar< T > > | inlinestatic |
| round\_style | std::numeric\_limits< stan::agrad::fvar< T > > | static |
| signaling\_NaN() | std::numeric\_limits< stan::agrad::fvar< T > > | inlinestatic |
| tinyness\_before | std::numeric\_limits< stan::agrad::fvar< T > > | static |
| traps | std::numeric\_limits< stan::agrad::fvar< T > > | static |


---

[ Stan Home Page ]

*© 2011–2014,
Stan Development Team.*
